# Comparative Analysis of Mass Spectral Similarity Measures on Peak Alignment for Comprehensive Two-Dimensional Gas Chromatography Mass Spectrometry

**DOI:** 10.1155/2013/509761

**Published:** 2013-09-16

**Authors:** Seongho Kim, Xiang Zhang

**Affiliations:** ^1^Biostatistics Core, Karmanos Cancer Institute, Wayne State University, Detroit, MI 48201, USA; ^2^Department of Chemistry, University of Louisville, Louisville, KY 40292, USA

## Abstract

Peak alignment is a critical procedure in mass spectrometry-based biomarker discovery in metabolomics. One of peak alignment approaches to comprehensive two-dimensional gas chromatography mass spectrometry (GC×GC-MS) data is peak matching-based alignment. A key to the peak matching-based alignment is the calculation of mass spectral similarity scores. Various mass spectral similarity measures have been developed mainly for compound identification, but the effect of these spectral similarity measures on the performance of peak matching-based alignment still remains unknown. Therefore, we selected five mass spectral similarity measures, cosine correlation, Pearson's correlation, Spearman's correlation, partial correlation, and part correlation, and examined their effects on peak alignment using two sets of experimental GC×GC-MS data. The results show that the spectral similarity measure does not affect the alignment accuracy significantly in analysis of data from less complex samples, while the partial correlation performs much better than other spectral similarity measures when analyzing experimental data acquired from complex biological samples.

## 1. Introduction

Metabolomics is the systematic study of metabolites found within cells and biological systems. It has emerged as the latest of the “omics” disciplines to decipher the complex time-related concentration, activity, and flux of metabolites in biological or clinical samples, offering a path to a wealth of information about a person's health.

Multiple analytical platforms such as liquid chromatography-mass spectrometry (LC-MS), gas chromatography-mass spectrometry (GC-MS), and nuclear magnetic resonance spectroscopy (NMR) have been used in metabolomics. Of these analytical platforms, the comprehensive two-dimensional gas chromatography coupled with mass spectrometry (GC×GC-MS) is a promising analytical platform in metabolomics for disease biomarker discovery [1–3]. This approach uses a short column as the second dimension GC column after the first dimension GC column which is the main analytical column. In general, these two columns have different stationary phases, and the first dimension column is operated at a lower temperature than the second dimension column. The difference of column temperature and the chromatography matrix enables the compounds coeluted from the first dimension column to be further separated in the second dimnsion column. The compounds separated in the second dimension column are directed to a mass spectrometry system for detection. The GC×GC-MS platform offers several advantages for analysis of complex samples, such as an order-of-magnitude increase in separation capacity, significant increase in signal-to-noise ratio and dynamic range, and improvement of mass spectral deconvolution and similarity matches [4, 5], providing more and accurate information about metabolite retention times and mass spectra.

In disease biomarker discovery, multiple samples from each biological cohort (disease or control) are usually collected to increase the statistical power, and each of these samples is preprocessed and analyzed on a high throughput analytical platform such as GC×GC-MS. Metabolic profiles obtained from these samples must then be aligned to compare the difference of abundance level of each compound between/among sample cohorts. The purpose of peak alignment is to recognize molecular features of the same metabolite occurring in different samples. Two alignment approaches have been developed: profile alignment and peak matching. The profile alignment uses the entire chromatographic data, that is, the raw instrumental data [6–9]. However, this approach aligns the GC×GC-MS data based on retention time alone, although the mass spectrum of fragment ions is readily available in the raw instrument data. Aligning metabolic profiles based on both retention time and mass spectrum can decrease the rate of false-positive alignment. In order to account for this fact, the peak matching approach was introduced. The raw instrument data, in this case, are first reduced into compound peak list, and the peak lists of multiple samples are then employed for alignment [10–15]. In this study, we examined the effects of mass spectral similarity measures on the performance of the peak matching-based alignment approach.

Several peak matching-based alignment algorithms have been developed, such as MSort [10], DISCO [11], mSPA [12], SWPA [13], and MbPA [14]. MSort is a two-step peak alignment using a distance window, while DISCO is a two-step peak alignment using a mass spectral similarity window. The algorithm mSPA employs a mixture similarity score to simultaneously evaluate both the retention time distance and the mass spectral similarity. SWPA performs peak alignment using Smith-Waterman local alignment algorithm. Of these methods, MbPA is the only model-based approach, which uses an empirical Bayes model and the posterior distribution for peak alignment. DISCO, SWPA, and MbPA can be applied to both homogeneous and heterogeneous data, while MSort and mSPA are able to align only for homogeneous data. The homogeneous data mean that all samples were analyzed under the identical GC×GC-MS experiment conditions, while the heterogeneous data refer to that experiment data were acquired under different experiment conditions. Most recently, Jeong et al. [15] proposed a post hoc analysis for peak alignment by incorporating the results of compound identification.

The retention time distance measure and the mass spectral similarity measure play a critical role in peak matching-based alignment. As for the retention time distance measure, MSort and DISCO use the Euclidean distance, while SWPA and MbPA use the rank of the Euclidean distance. In particular, mSPA investigated the effect of the four different distance measures, including Euclidean distance, Maximum (also known as Chebyshev) distance, Manhattan distance, and Canberra distance, on peak alignment and concluded that the Canberra distance is a promising distance measure for peak alignment. In case of the mass spectral similarity measure, MSort, DISCO, and SWPA use Pearson's correlation, while mSPA and MbPA use the cosine correlation (also known as dot product).

The mass spectral similarity measure is the key to compound identification in metabolomics, and is fulfilled by matching experimental mass spectra to mass spectra stored in a reference library. Various mass spectral similarity measures have been developed including cosine correlation [16], composite similarity [16], probability-based matching system [17], Hertz et al. similarity index [18], normalized Euclidean distance [19], absolute value distance [19], and wavelet and Fourier transforms-based composite measures [20]. Later, Kim et al. [21] developed partial and semipartial correlation-based similarity measures and showed that their similarity measures perform better than the dot product and its composite versions, including wavelet and Fourier transforms-based composite measures. 

Although both the compound identification and the peak alignment use mass spectra and the effect of mass spectral similarity measures on compound identification has been studied, the effect of the different mass spectral similarity measures on the performance of peak alignment still remains unknown. Therefore, the objective of this work was to compare the effects of five mass spectral similarity measures, cosine correlation, Pearson's correlation, Spearman's correlation, partial correlation, and part (also known as semipartial) correlation, on peak alignment. For ease of comparison, we selected the peak alignment algorithm mSPA since it includes various peak alignment approaches and the homogeneous data are more practically applicable.

The remaining of the paper is organized as follows.  Section 2 contains a review of mSPA and a detailed description of five mass spectral similarity measures. In Section 3, the selected mass spectral similarity measures were applied to experimental GC×GC-MS data to investigate the effect of the mass spectral similarity measures on peak alignment using mSPA. Finally, Section 4 provides some discussion and is closed with conclusions. 

## 2. Method and Material

Let *R* = {*r*_1_, *r*_2_,…, *r*_*m*_} be the peak list of a reference chromatogram and *T* = {*t*_1_, *t*_2_,…, *t*_*n*_} the peak list of a target chromatogram, where *r*_*i*_ and *t*_*j*_ (1 ≤ *i* ≤ *m*, 1 ≤ *j* ≤ *n*) are composed of its first and second dimension retention times, (*r*_*i*,1_, *r*_*i*,2_) and (*t*_*j*,1_, *t*_*j*,2_), respectively, as well as its mass spectrum, *X*_*r*_*i*__ and *X*_*t*_*j*__, respectively. Note that the mass spectrum *X*_*a*_ is a vector of intensities for the peak *a*, such as *X*_*a*_ = (*x*_1_, *x*_2_,…, *x*_*g*_), where *g* is the total number of mass-to-charge ratio (*m/z*). We call each peak in the reference peak list a reference peak and a peak in the target peak list a target peak. The distance and the similarity refer to the retention times and the mass spectral information, respectively. All the statistical analyses and simulations were performed using a statistical package R (R Development Core Team).

### 2.1. Review of mSPA

The peak alignment R package *mSPA* [12] provides five peak alignment algorithms for users (http://mrr.sourceforge.net/). The five peak alignment algorithms are PAD, PAS, SW-PAD, DW-PAS, and PAM. Here PAD is a peak alignment procedure using solely the peak distance without window, and PAS performs the peak alignment based on the spectral similarity without window. SW-PAD and DW-PAS are window-based peak alignments. SW-PAD stands for the peak alignment with a similarity-based window, and DW-PAS aligns peaks using a distance-based window. Kim et al. [12] further developed a mixture similarity measure (*M*_*d*_). That is, the mixture similarity score between a target peak *t*_*j*_ and a reference peak *r*_*h*_ is defined by

(1) $$\begin{array}{c} M_{d}\left(t_{j},r_{i}\right)=\frac{w}{1+D_{d}\left(t_{j},r_{i}\right)}+\left(1-w\right)\cdot S\left(t_{j},r_{i}\right), \end{array}$$

where *w*  (0 ≤ *w* ≤ 1) is a mixture weight factor, *S*(*t*, *r*) and *D*_*d*_(*t*, *r*) are a spectral similarity score and a distance measure between two peaks *t* and *r*, respectively. PAM is the peak alignment method using this mixture similarity without any window. The main difference of PAM over other approaches is the ability to use both the retention time distance and the mass spectral similarity at the same time without window. In addition, an optimization-based peak alignment, OP-PAM, is also incorporated in mSPA. OP-PAM is the optimal version of PAM and optimizes the mixture weight w and the distance measure. For further details refer to Kim et al. [12].

mSPA uses the cosine correlation as the main mass spectral similarity measure, although a user can choose Pearson's correlation coefficient as an option. mSPA also includes four distance measures, such as Euclidean (*D*_1_), Maximum (*D*_2_), Manhattan (*D*_3_), and Canberra (*D*_4_). Kim et al. [12] showed that Canberra distance performs the best among them. However, it still remains unknown which similarity measure performs better for peak alignment.

### 2.2. Similarity Measures

In this study, we selected five similarity measures, cosine correlation, Pearson's correlation, Spearman's correlation, partial correlation, and part correlation. Since all the existing peak matching-based approaches use either the cosine correlation or Pearson's correlation, we chose these two mass spectral similarity measures. Spearman's correlation was considered because it is a nonparametric measure. The partial and the part correlations were selected because of their best performance in compound identification [21].

#### 2.2.1. Cosine Correlation (Dot Product)

The cosine correlation [16], which is also known as the dot product, is used to obtain the cosine of the angle between two sequences of intensities, *X* = (*x*_*i*_)_*i*=1,…,*g*_ and *Y* = (*y*_*i*_)_*i*=1,…,*g*_, where *g* is the total number of *m/z* values. It is defined as

(2) $$\begin{array}{c} c_{XY}=C\left(X,Y\right)=\;\frac{X\circ Y}{||X||\cdot ||Y||}, \end{array}$$

where *X*∘*Y* = ∑_*i*=1_^*g*^ *x*_*i*_ *y*_*i*_ and $||X||=\sqrt{\sum_{i=1}^{g}x_{i}^{2}}$. Note that *c*_*XY*_ ranges between −1 and 1, and it is always nonnegative if *X* and *Y* are nonnegative intensities.

#### 2.2.2. Pearson's and Spearman's Correlations

Pearson's correlation between two sequences of intensities, *X* = (*x*_*i*_)_*i*=1,…,*g*_ and *Y* = (*y*_*i*_)_*i*=1,…,*g*_, is the covariance of the two sequences divided by the product of the standard deviations and is defined by

(3) $$\begin{array}{c} r_{XY}=\text{Corr}\left(X,Y\right)=\frac{\text{Cov}\left(X,Y\right)}{\sqrt{\mathrm{Var}\left(X\right)}\sqrt{\mathrm{Var}\left(Y\right)}}, \end{array}$$

where Cov(*X*, *Y*) is the covariance between *X* and *Y* and Var⁡(*X*) is the variance of *X*. Spearman's correlation between *X* and *Y*, *ρ*_*XY*_, is a nonparametric version of Pearson's correlation and is defined as Pearson correlation coefficient between the ranks of two sequences of intensities.

#### 2.2.3. Partial and Part (Semipartial) Correlations

The partial correlation is the association between two random variables after removing the effect of other random variables, while the part correlation removes the effect of other random variables only for one random variable [21]. Consider a partitioned random vector (*X*, *Y*) where *X* and *Y* = (*Y*_1_, *Y*_2_,…, *Y*_*h*_) are one-dimensional random variables and an *h*-dimensional random vector, respectively. Then the partial correlation *r*_*XY*_*i*_∣*Y*^(*i*)^_ between *X* and *Y*_i_ given *Y* ^(*i*)^ = (*Y*_1_,…, *Y*_*i*−1_, *Y*_*i*+1_,…, *Y*_*h*_) is defined by the correlation between the residuals *R*_*X*∣*Y*^(*i*)^_ and *R*_*Y*_*i*_∣*Y*^(*i*)^_ and is represented by

(4) $$\begin{array}{c} r_{{XY}_{i}\;|\;Y^{\left(i\right)}}=\text{Corr}\left(R_{X\;|\;Y^{\left(i\right)}},R_{Y_{i}\;|\;Y^{\left(i\right)}}\right), \end{array}$$

where *R*_*X*∣*Y*^(*i*)^_ and *R*_*Y*_*i*_∣*Y*^(*i*)^_ are the residuals of the linear regression of *X* and *Y*_*i*_ on *Y* ^(*i*)^, respectively. 

The semipartial correlation *r*_*X*(*Y*_*i*_∣*Y*^(*i*)^)_ between *X* and *Y*_i_ with *Y* ^(*i*)^ is the correlation between the random variable *X* and *R*_*Y*_*i*_|*Y*^(*i*)^_ and is represented by

(5) $$\begin{array}{c} r_{X(Y_{i}\;|\;Y^{\left(i\right)})}=\text{Corr}\left(X,R_{Y_{i}\;|\;Y^{\left(i\right)}}\right). \end{array}$$

In general, *r*_*X*(*Y*_*i*_∣*Y*^(*i*)^)_ ≠ *r*_*Y*_*i*_(*X*∣*Y*^(*i*)^)_ and *r*_*X*(*Y*_*i*_∣*Y*^(*i*)^)_ = *r*_*Y*_*i*_(*X*∣*Y*^(*i*)^)_ if *X* and *Y*_*i*_ are independent of *Y* ^(*i*)^. If *X* and *Y*_*i*_ are independent of *Y* ^(*i*)^, all the three correlations, Pearson's, partial, and part correlations, are theoretically exactly similar to each other, that is, *r*_*XY*_*i*__ = *r*_*XY*_*i*_∣*Y*^(*i*)^_ = *r*_*X*(*Y*_*i*_∣*Y*^(*i*)^)_. In the context of peak alignment, *X* is the mass spectrum of a target peak and *Y* is the vector of all the mass spectra of the reference peak list.

It is known that the partial correlation can be derived by the inverse of the covariance matrix [22], so does the part correlation. In the context of partial and part correlations, each peak represents a random variable and the intensities of each *m/z* value correspond to the observed samples, resulting in the number of peaks being equal to the number of variables and the number of *m/z* values being equal to the sample size. However, the number of peaks often exceeds the number of *m/z* values in case of real biological data, resulting in a high-dimensionality problem. This causes the singularity of the inverse covariance matrices between two peak lists. To avoid the singularity problem, we adopted the two-step approach developed by Kim et al. [21]. Namely, we first reduced the number of peaks for the calculation of the partial and the part correlations by considering only the peaks that have the first *q* highest similarity scores obtained by Pearson's correlation. Then the partial and the part correlations were computed only for these *q* peaks. Given the rank *q*, the two-step partial and part correlations are defined by, respectively,

(6) $$\begin{array}{c} r_{{XY}_{i}\;|\;Y^{\left(i,q\right)}}=\text{Corr}\left(R_{X\;|\;Y^{\left(i,q\right)}},R_{Y_{i}\;|\;Y^{\left(i,q\right)}}\right),\\ r_{{X(Y}_{i}\;|\;Y^{\left(i,q\right)})}=\text{Corr}\left(X,R_{Y_{i}\;|\;Y^{\left(i,q\right)}}\right), \end{array}$$

where *Y* ^(*i*,*q*)^ = {*Y*_*j*_ | Rank(*r*_*XY*_*j*__) ≤ *q*, *Y*_*j*_ ∈ *Y* ^(*i*)^} and Rank(*r*_*XY*_*j*__) is the rank of the similarity score *r*_*XY*_*j*__ in descending order. In this study, (4) and (5) were applied to a mixture of 76 compound standards, and a biological data set employed (6) to avoid the singularity of the covariance matrix. We used 10 different ranks between 3 and 100 for *q*, which are 3, 5, 7, 10, 15, 20, 30, 50, 70, and 100. The R package *ppcor* was used to compute the partial and the part correlations. 

### 2.3. GC×GC Data Sets

**Figure 1:**
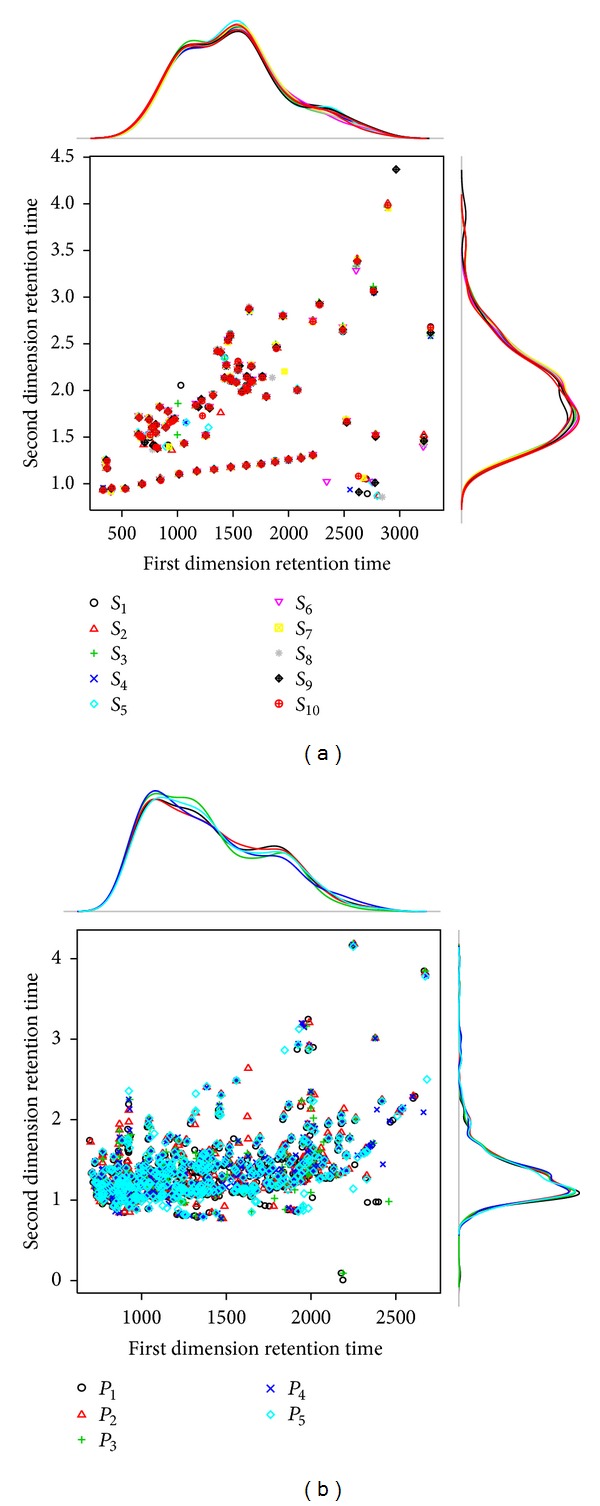
The chromatogram of GC×GC/TOF-MS datasets. The estimated kernel density plots are of the first and second dimension retention times for each of Data I (a) and Data II (b).

For a fair comparison with mSPA, we used the same data as those of mSPA, which are a mixture of 76 compound standards and a set of real biological samples extracted from rat plasma. A mixture of 76 compound standards is composed of 10 GC×GC-MS data sets (S1–S10), and the rat plasma sample consists of five GC×GC-MS data sets (P1–P5). For a more detailed description of the data, please refer to Wang et al. [11]. We call the mixture of 76 compound standards Data I, which has 10 data sets, and the rat plasma data set Data II, which has 5 data sets. Theoretically, one peak should be generated for each compound after peak picking. Multiple peaks, however, are usually detected for one compound by the spectral deconvolution software such as ChromaTOF, which will generate a set of peak lists. Therefore, we merged the multiple peaks by peak area. In other words, we selected the peak with the largest peak area among the multiple peaks having the same compound name. The number of peaks before and after peak merging is summarized in Table 1. The chromatograms and the densities of the first and the second dimension retention times of Data I and Data II are depicted in Figure 1. Note that the data and source code are available at http://mrr.sourceforge.net/.

### 2.4. Performance Criteria

The true positive rate (TPR), the false positive rate (FPR), the positive predictive value (PPV), the F1 score, and the area under receiver operating characteristic (ROC) curve are used to compare the performance of each similarity measure in peak alignment. Let *R* = {*r*_1_, *r*_2_,…*r*_*s*_, *r*_*s*+1_,…, *r*_*m*_} be the peak list of a reference chromatogram and *T* = {*t*_1_, *t*_2_,…, *t*_*s*_, *t*_*s*+1_,…, *t*_*n*_} the peak list of a target chromatogram. Suppose there are *s* true peak pairs {(*r*_1_, *t*_1_), (*r*_2_, *t*_2_),…, (*r*_*s*_, *t*_*s*_)} and *u* peak pairs are matched by a certain peak alignment, where *s*, *u* ≤ min⁡(*n*, *m*). Define the number of true positive (TP) as the number of true positive peak pairs, which is less than or equal to min⁡⁡(*s*, *u*). Then the number of false positive (FP) becomes *u*−TP, the number of false negative (FN) becomes *s*−TP, and the number of true negative (TN) becomes *m* · *n* − *s* − FP. As a result, TPR, FPR, PPV, and F1 score are defined by

(7) $$\begin{array}{c} \text{TPR}=\frac{\text{TP}}{\text{TP}+\text{FN}}=\frac{\text{TP}}{s},\\ \text{FPR}=\frac{\text{FP}}{\text{TN}+\text{FP}}=\frac{u-\text{TP}}{m\cdot n-s},\\ \text{PPV}=\frac{\text{TP}}{\text{TP}+\text{FP}}=\frac{\text{TP}}{u},\\ F_{1}=\frac{2\cdot \text{TPR}\cdot \text{PPV}}{\text{TPR}+\text{PPV}}=\frac{2\text{TP}}{s+u}. \end{array}$$

The area under ROC curve (AUC) was further calculated after ROC was created by plotting between TPR and FPR according to given cut-off values using the methods in [23, 24].

## 3. Results

We evaluated the effect of the five spectral similarity measures on peak alignment using mSPA. As mentioned before, mSPA provides five different peak alignment methods including an optimal version. In this study, we focused only on the following four methods: PAS, DW-PAS, SW-PAD, and PAM, since we were interested in the effect of the mass spectral similarity measures. Therefore, these four peak matching alignment approaches were applied to Data I and Data II using mSPA, with the five different similarity measures, the cosine correlation, Pearson's correlation, Spearman's correlation, the partial correlation, and the part correlation. 

**Figure 2:**
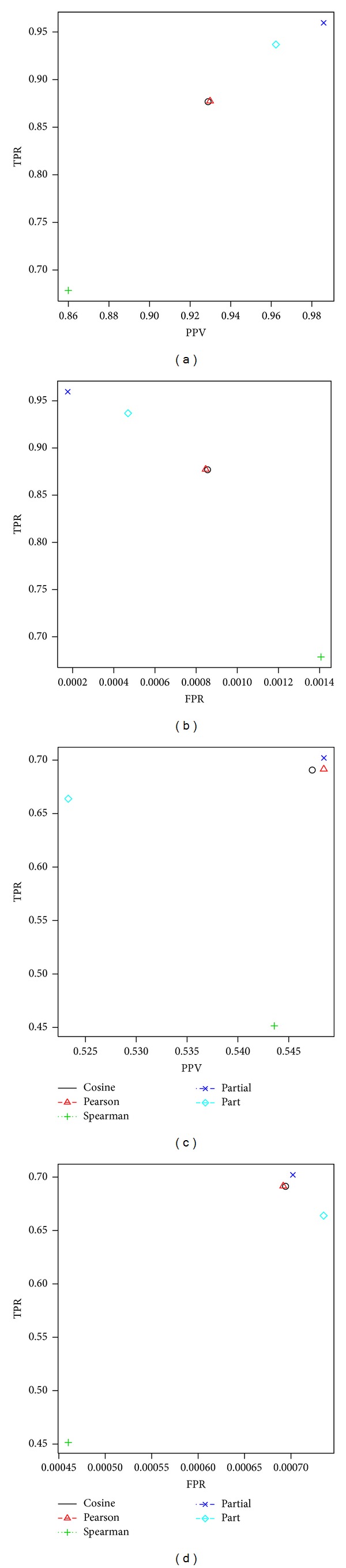
The results of peak alignment using PAS. (a) and (b) are for Data I and (c) and (d) are for Data II. The plots of PPV versus TPR are in (a) and (c), and the plots of FPR versus TPR are in (b) and (d).

Figure 2 displays the plots of PPV versus TPR and FPR versus TPR when PAS was applied to Data I and II. In the PPV versus TPR plot, a method is better as it is closer to the point (1,1), while the FPR versus TPR plot represents that a method is better as it is close to the point (0,1). It is worth reminding that PAS is a peak alignment solely based on the mass spectral similarity score without using the retention time distance. Spearman's correlation performs the worst for both Data I (75.52%) and Data II (49.31%) in terms of F1 scores, while the partial correlation performs the best (97.24% and 61.58% for Data I and II, resp.), as can be seen in Tables 2 and 3. Interestingly, the partial correlation performs better than the part correlation.

**Figure 3:**
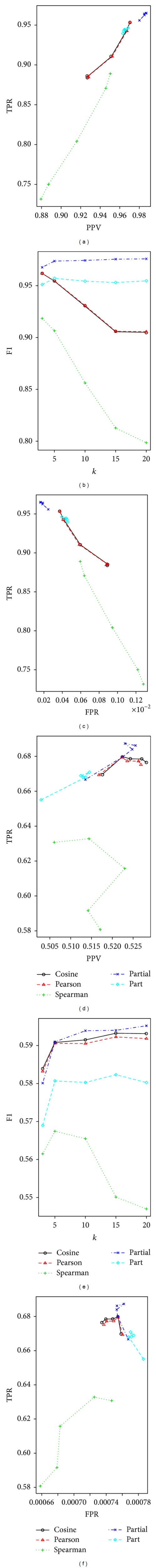
The results of peak alignment using DW-PAS. (a)–(c) are for Data I and (d)–(f) are for Data II. The plots of PPV versus TPR are in (a) and (d), the plots of F1 scores are in (b) and (e), and the plots of FPR versus TPR are in (c) and (f).

The method DW-PAS is a peak alignment method with a distance-based window. In this case, a user is required to set a threshold for the distance-based window, which is the rank *k* of the retention time distance. The five different ranks, 3, 5, 10, 15, and 20, were used. The plots of PPV versus TPR, F1 scores, and FPR versus TPR (ROC) are shown in Figure 3. Likewise, Spearman's correlation performs the worst regardless of the rank *k* and the dataset. As the rank *k* increases, the F1 scores of the partial and the part correlations generally increase, while the F1 scores of the cosine, Pearson's, and Spearman's correlations decrease, in case of Data I (Figure 3(b)). On the other hand, in case of Data II, the F1 scores of the cosine, Pearson's, the partial, and the part correlations increase as the rank *k* increases, while Spearman's correlation decreases. Overall, the partial correlation performs the best for both Data I (97.59%) and Data II (59.52%) in terms of F1 scores, as shown in Tables 2 and 3.

**Figure 4:**
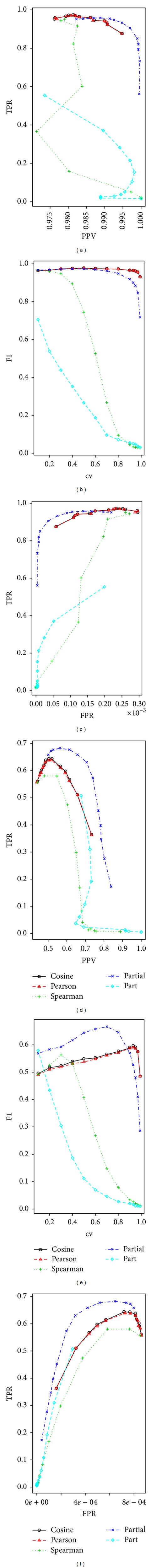
The results of peak alignment using SW-PAD. (a)–(c) are for Data I and (d)–(f) are for Data II. The plots of PPV versus TPR are in (a) and (d), the plots of F1 scores are in (b) and (e), and the plots of FPR versus TPR are in (c) and (f).

Figure 4 shows the results of SW-PAD. This method requires a mass spectral similarity-based window as well as a cut-off value of the similarity (0 ≤ *ρ* ≤ 1). In this study, we used 13 values between 0.1 and 0.99 for *ρ*. The F1 scores of the part and Spearman's correlations are much sensitive to the cut-off value *ρ* than these of other correlations in Figures 4(b) and 4(e). In case of Data I, Pearson's correlation (97.68%) with Canberra distance performs the best among them in terms of F1 score, while the F1 score (66.65%) of the partial correlation with Manhattan distance is the highest when Data II is applied, as can be seen in Tables 2 and 3. 

**Figure 5:**
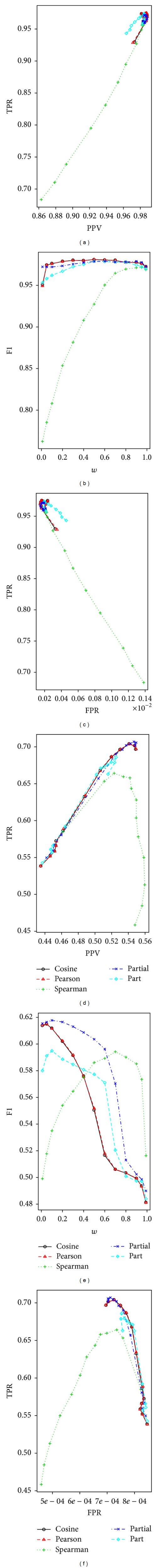
The results of peak alignment using PAM. (a)–(c) are for Data I and (d)–(f) are for Data II. The plots of PPV versus TPR are in (a) and (d), the plots of F1 scores are in (b) and (e), and the plots of FPR versus TPR are in (c) and (f).

The PAM aligns peak lists using a mixture similarity score of the retention time distance and the mass spectral similarity. In this case, a user needs to set up the mixture weight (0 ≤ *w* ≤ 1). If *w* is close to zero, the mass spectral similarity plays a much more important role in peak alignment than the retention time distance does, while the retention time distance drives the peak alignment if *w* close to one, as can be seen in (1). We used 13 values between 0.01 and 0.99 for *w*. Similar to the other peak alignment approaches, Spearman's correlation performs the worst among them in terms of F1 scores, as shown in Figures 5(b) and 5(e). As for Data I, all the correlations except for Spearman's correlation are less sensitive to the mixture weight *w*, while the F1 scores of all the correlations are more sensitive to the weight *w* in case of Data II. In Data I, the highest F1 score (98.12%) occurred when Pearson's correlation used, and, as for Data II, the partial correlation had the highest F1 score (61.78%), as shown in Tables 2 and 3.

Overall, Pearson's correlation with PAM performs the best in terms of F1 score for Data I (98.12%), and the partial correlation with SW-PAD performs the best for Data II (66.65%), as can be seen in Tables 2 and 3. Interestingly, the partial correlation always has the highest AUC across the approaches in case of Data I. More detailed F1 scores and AUCs for each of the distance measures and the datasets can be found in the Supplementary Material Tables S1–S8 (Supplementary Material available online at http://dx.doi.org/10.1155/2013/509761).

## 4. Discussion and Conclusions

When the less dense data such as Data I are applied, the effect of the mass spectral similarity measures on the performance of peak alignment is small since the retention time distance dominates the performance of peak alignment. In fact, F1 scores of all the mass spectral similarity measures except for the Spearman's correlation are not significantly different from each other when PAM is applied to Data I, as shown in Table 2. On the other hand, when analyzing more complicated data such as Data II, the mass spectral similarity measures play a critical role in obtaining a better performance of peak alignment. As can be seen in Table 3, the F1 score of the partial correlation with SW-PAD is significantly different from those of other methods. Furthermore, all the peak alignment approaches perform the best when the partial correlation is employed, indicating that the effect of the mass spectral similarity measures on alignment is critical and we should consider the partial correlation to achieve a better performance.

In case that the mass spectral similarity measures were compared to each other in terms of accuracy of compound identification, the part correlation performed the best although its performance was comparable to that of the partial correlation [21]. Different from compound identification, the partial correlation performs significantly better than the part correlation in peak alignment. For example, we can see this from the results of PAS listed in Tables 2 and 3. Interestingly, when the more dense data are used, the performance of the part correlation with PAS becomes worse than those of the cosine and Pearson's correlations. This may be because the characteristics of the experimental data are different between compound identification and peak alignment. Namely, in compound identification, the query mass spectra are generated from the experimental conditions typically different from that of the reference library mass spectra. Therefore, the effect of the reference library mass spectra is ignorable so that the part correlation performs the best. On the other hand, the peak alignment here uses the homogeneous data which are generated from the similar experimental conditions, resulting in that the partial correlation performs the best.

**Figure 6:**
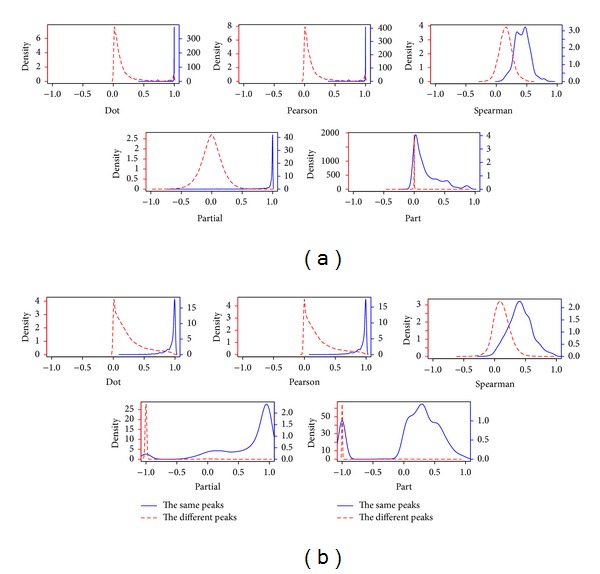
The distributions of similarity scores from the same and the different peaks. The blue solid lines and the red dotted lines represent the distributions of the similarity scores between the same peaks and between the different peaks, respectively. The left *y*-axis is scaled for the different peaks and the right *y*-axis is scaled for the same peaks. (a) and (b) are for Data I and Data II, respectively.

To further investigate this difference of the five similarity measures, we plotted the distributions of the five similarity scores from the same peaks as well as from the different peaks for Data I and Data II, as shown in Figure 6. In an ideal case, the distribution of the same peaks (the blue solid line) should be close to 1, and the distribution of the different peaks (the red dotted line) should be close to either 0 (for the cosine correlation) or −1 (for other similarity measures). We can see that the distributions of the partial correlation are clearly separated among the five mass spectral similarity measures (including the part correlation), explaining why the partial correlation with PAS performs the best in terms of F1 scores. In addition, the distributions of the cosine and Pearson's correlations have the very similar trends to each other for both Data I and Data II. In fact, this is consistent with the result of the comparison analysis of Liu et al. [25], in which Pearson's correlation coefficient is most robust, but the difference between the dot product and Pearson's correlation coefficient is subtle.

Another point to consider is that SW-PAD with the partial correlation is the best approach in case of Data II, while PAM is the best approach with Data I. In fact, the F1 score of SW-PAD with the partial correlation is improved up to 5%, compared to that of PAM with the partial correlation in case of Data II. This may be because more peaks in Data II have similar mass spectral information although they are generated from the different compounds. For example, the cut-off value *ρ* of SW-PAD with the partial correlation is much larger in Data II than that in Data I (Tables 2 and 3).

In conclusion, as for the less dense data such as Data I, PAM with any one of the cosine, the Pearson's, and the partial correlations will give us a better performance of peak alignment, while SW-PAD with the partial correlation will perform the best in case of the more dense data, such as the data acquired from real biological samples. 

## Supplementary Material

This Supplementary Material includes Tables S1-S8 with more detailed F1 scores and AUCs for each of the distance measures.

## Figures and Tables

**Table 1:** The summary of GC×GC/TOF-MS datasets. The numbers of peaks before and after peak merging are calculated for each dataset.

Run ID	Data I	S1	S2	S3	S4	S5	S6	S7	S8	S9	S10
The number of peaks	Before	180	186	161	151	151	145	172	163	168	174
	After	78	76	76	75	74	73	74	76	77	75

Run ID	Data II	P1	P2	P3	P4	P5					

The number of peaks	Before	759	733	694	727	661					
	After	466	456	436	452	418					

**Table 2:** F1 score and AUC of each peak alignment method for Data I.

		Cosine	Pearson's	Spearman's	Partial	Part
F1 (%)	PAS	90.23*	90.30	75.82	***97.24 ***	94.93
		(0.69)^#^	(0.68)	(0.80)	***(0.18)***	(0.23)
	DW-PAS	96.18	96.18	91.84	***97.59***	95.72
		(0.37)	(0.37)	(0.51)	***(0.25)***	(0.36)
	*k* ^$^	3	3	3	***20***	5
	Distance**	E	E	E	E, Mx, Mh	Mx
	SW-PAD	97.66	***97.68 ***	96.47	97.31	70.52
		(0.26)	***(0.26)***	(0.27)	(0.28)	(0.39)
	*ρ* ^##^	0.5	***0.5***	0.1	0.4	0.1
	Distance	C	C	C	E, Mh	E, Mx, Mh
	PAM	98.10	***98.12 ***	97.15	97.89	97.91
		(0.20)	***(0.20)***	(0.26)	(0.21)	(0.24)
	*W* ^$$^	0.5	***0.5***	0.95	0.6	0.5
	Distance	C	C	C	C	C

AUC (%)	PAS	93.82	93.83	83.85	***97.97***	96.81
	DW-PAS	94.25	94.26	86.55	***97.79***	96.95
	SW-PAD	97.59	97.82	97.16	***97.99***	77.68
	PAM	96.47	96.42	84.16	***98.10***	97.15

*Mean (%); ^#^standard error (%); ^$^the cut-off rank; **the distance measure E, Mx, Mh, and C stand for Euclidean, Maximum, Manhattan, and Canberra distances, respectively; ^##^the cut-off similarity score; ^$$^the weight factor of the mixture similarity score. The numbers in bold and italic indicate the maximum for each of the peak alignment methods.

**Table 3:** F1 score and AUC of each peak alignment method for Data II.

		Cosine	Pearson's	Spearman's	Partial	Part
F1 (%)	PAS	61.09*	61.17	49.31	***61.58***	58.52
		(0.31)^#^	(0.30)	(0.25)	***(0.87)***	(0.85)
	Rank				***30***	5
	DW-PAS	59.32	59.23	56.74	***59.52***	58.23
		(0.33)	(0.34)	(0.32)	***(0.95)***	(1.02)
	*k* ^$^	15	15	5	***20***	15
	q**				***50***	15
	Distance^##^	C	C	C	C	C
	SW-PAD	59.55	59.16	56.32	***66.65***	57.96
		(0.41)	(0.36)	(0.37)	***(0.77)***	(0.75)
	*ρ* ^$$^	0.93	20	0.3	***0.7***	0.1
	*q*				***50***	100
	Distance	Mh	Mx	Mh	Mh	Mh
	PAM	61.48	61.51	59.42	***61.78***	60.19
		(0.31)	(0.33)	(0.36)	***(0.91)***	(1.00)
	w***	0.05	0.05	0.7	***0.1***	0.5
	*q*				***30***	3
	Distance	E, Mx, Mh	Mx	C	E, Mx, Mh	Mh

AUC (%)	PAS	84.53	84.55	72.55	***85.07***	83.16
	DW-PAS	***83.45***	83.43	81.50	83.30	82.71
	SW-PAD	78.06	77.81	77.77	***82.90***	75.35
	PAM	76.89	76.89	***79.22***	77.45	77.18

*Mean (%); ^#^standard error (%); ^$^the cut-off rank; **the rank for the two-step partial and part correlations; ^##^the distance measure E, Mx, Mh, and C stand for Euclidean, Maximum, Manhattan, and Canberra distances, respectively; ^$$^the cut-off similarity score. ***the weight factor of the mixture similarity score; The numbers in bold and italic indicate the maximum for each of the peak alignment methods.
