# Supplementary material for: Comparative Analysis of Mass Spectral Similarity Measures on Peak Alignment for Comprehensive Two-Dimensional Gas Chromatography Mass Spectrometry
Source: Comput Math Methods Med. 2013 Sep 16;2013:509761. doi: 10.1155/2013/509761 (PMC3787630; doi:10.1155/2013/509761)
Supplement: Supplementary file 1 — This Supplementary Material includes Tables S1-S8 with more detailed F1 scores and AUCs for each of the distance measures. [file 509761.f1.pdf]

Comparative Analysis of Mass Spectral Similarity Measures on Peak Alignment for Two-dimensional Gas Chromatography Mass Spectrometry

Seongho Kim[1,*] and Xiang Zhang[2]

[1]Biostatistics Core, Karmanos Cancer Institute, School of Medicine, Wayne State University, Detroit, MI

[2]Department of Chemistry, University of Louisville, Louisville, KY

[*], Corresponding author's email address: kimse@karmanos.org

Supplementary Information

Table S1. F1 score and AUC of each peak alignment method using Euclidean distance for Data I.

		Cosine	Pearson's	Spearman's	Partial	Part
F1 (%)	PAS	90.23* (0.69)#	90.30 (0.68)	75.82 (0.80)	***97.24 (0.18)***	94.93 (0.23)
	DW-PAS	96.18 (0.37)	96.18 (0.37)	91.84 (0.51)	***97.59 (0.25)***	95.64 (0.36)
	$k^{\$}$	3	3	3	***20***	5
	SW-PAD	97.36 (0.25)	***97.36 (0.24)***	93.61 (0.51)	97.31 (0.28)	70.52 (0.39)
	ρ^{**}	0.8	***0.6***	0.1	0.4	0.1
	PAM	***97.80 (0.23)***	***97.80 (0.23)***	94.19 (0.42)	97.65 (0.25)	97.00 (0.26)
	$w^{\#\#}$	***0.3***	***0.3***	0.6	0.3	0.1
AUC (%)	PAS	93.82	93.83	83.85	***97.97***	96.81
	DW-PAS	94.25	94.26	86.55	***97.79***	96.95
	SW-PAD	96.67	96.67	95.48	***97.58***	77.67
	PAM	95.35	95.35	85.64	***95.41***	95.05

***, Mean (%); #, Standard error (%); $, the cut-off rank; **, the cut-off similarity score; ##, the weight factor of the mixture similarity score; The numbers in bold and italic indicate the maximum for each of the peak alignment methods.**

Table S2. F1 score and AUC of each peak alignment method using Maximum distance for Data I.

		Cosine	Pearson's	Spearman's	Partial	Part
**F1 (%)**	**PAS**	90.23* (0.69)#	90.30 (0.68)	75.82 (0.80)	***97.24 (0.18)***	94.93 (0.23)
	**DW-PAS**	96.14 (0.37)	96.14 (0.37)	91.81 (0.51)	***97.59 (0.25)***	95.72 (0.36)
	k$	3	3	3	***20***	5
	**SW-PAD**	***97.36 (0.25)***	97.34 (0.26)	93.56 (0.39)	97.26 (0.27)	70.52 (0.39)
	ρ**	***0.8***	0.8	0.3	0.4	0.1
	**PAM**	***97.80 (0.23)***	***97.80 (0.23)***	94.19 (0.42)	97.65 (0.25)	96.99 (0.26)
	w##	***0.3***	***0.3***	0.6	0.3	0.1
**AUC (%)**	**PAS**	93.82	93.83	83.85	***97.97***	96.81
	**DW-PAS**	94.28	94.30	86.55	***97.79***	96.95
	**SW-PAD**	95.64	95.73	94.47	***97.16***	77.68
	**PAM**	95.35	95.35	85.64	***95.41***	95.04

***, Mean (%); #, Standard error (%); $, the cut-off rank; **, the cut-off similarity score; ##, the weight factor of the mixture similarity score; The numbers in bold and italic indicate the maximum for each of the peak alignment methods.**

Table S3. F1 score and AUC of each peak alignment method using Manhattan distance for Data I.

		Cosine	Pearson's	Spearman's	Partial	Part
**F1 (%)**	**PAS**	90.23* (0.69)#	90.30 (0.68)	75.82 (0.80)	***97.24 (0.18)***	94.93 (0.23)
	**DW-PAS**	96.04 (0.39)	96.04 (0.39)	91.83 (0.51)	***97.59 (0.25)***	95.64 (0.36)
	k$	3	3	3	***20***	5
	**SW-PAD**	97.36 (0.25)	***97.36 (0.24)***	93.80 (0.47)	97.31 (0.28)	70.52 (0.39)
	ρ**	0.8	***0.6***	0.1	0.4	0.1
	**PAM**	***97.80 (0.23)***	***97.80 (0.23)***	94.30 (0.41)	97.65 (0.25)	96.99 (0.26)
	w##	***0.3***	***0.3***	0.6	0.3	0.1
**AUC (%)**	**PAS**	93.82	93.83	83.85	***97.97***	96.81
	**DW-PAS**	94.25	94.26	86.55	***97.79***	96.95
	**SW-PAD**	96.84	96.84	95.59	***97.58***	77.68
	**PAM**	95.35	95.35	85.64	***95.41***	95.05

***, Mean (%); #, Standard error (%); $, the cut-off rank; **, the cut-off similarity score; ##, the weight factor of the mixture similarity score; The numbers in bold and italic indicate the maximum for each of the peak alignment methods.**

Table S4. F1 score and AUC of each peak alignment method using Canberra distance for Data I.

		Cosine	Pearson's	Spearman's	Partial	Part
**F1 (%)**	**PAS**	90.23[*]	90.30	75.82	***97.24***	94.93
		(0.69)[#]	(0.68)	(0.80)	***(0.18)***	(0.23)
	**DW-PAS**	90.54	90.60	86.85	***97.57***	96.54
		(0.67)	(0.66)	(0.55)	***(0.22)***	(0.29)
	k[$]	20	20	3	***10***	3
	**SW-PAD**	97.66	***97.68***	96.47	97.30	70.50
		(0.26)	***(0.26)***	(0.27)	(0.25)	(0.40)
	ρ[**]	0.5	***0.5***	0.1	0.4	0.1
	**PAM**	98.10	***98.12***	97.15	97.89	97.91
		(0.20)	***(0.20)***	(0.26)	(0.21)	(0.24)
	w[##]	0.5	***0.5***	0.95	0.6	0.5
**AUC (%)**	**PAS**	93.82	93.83	83.85	***97.97***	96.81
	**DW-PAS**	93.92	93.93	85.51	***98.24***	96.92
	**SW-PAD**	97.59	97.82	97.16	***97.99***	77.65
	**PAM**	96.47	96.42	84.16	***98.10***	97.15

[*], Mean (%); [#], Standard error (%); [$], the cut-off rank; [**], the cut-off similarity score; [##], the weight factor of the mixture similarity score; The numbers in bold and italic indicate the maximum for each of the peak alignment methods.

Table S5. F1 score and AUC of each peak alignment method using Euclidean distance for Data II.

		Cosine	Pearson's	Spearman's	Partial	Part
**F1 (%)**	**PAS**	61.09* (0.31)#	61.17 (0.30)	49.31 (0.25)	***61.58 (0.87)***	58.52 (0.85)
	**Rank**				***30***	5
	**DW-PAS**	***59.15 (0.35)***	59.09 (0.35)	56.72 (0.32)	59.12 (1.09)	57.54 (1.05)
	k$	***20***	20	10	20	20
	**q****				30	3
	**SW-PAD**	59.47 (0.42)	59.30 (0.39)	55.97 (0.39)	***66.60 (0.79)***	57.96 (0.78)
	ρ##	0.93	0.93	0.3	***0.7***	0.1
	**q**				***50***	100
	**PAM**	61.48 (0.31)	61.51 (0.33)	59.29 (0.30)	***61.78 (0.91)***	60.19 (1.00)
	w$$	0.05	0.05	0.1	***0.1***	0.5
	**q**				***30***	3
**AUC (%)**	**PAS**	84.53	84.55	72.55	***85.07***	83.16
	**DW-PAS**	***81.50***	81.50	80.26	81.23	80.59
	**SW-PAD**	78.04	78.36	77.71	***82.81***	75.35
	**PAM**	76.89	76.89	76.77	***77.45***	77.16

***, Mean (%); #, Standard error (%); $, the cut-off rank; **, the rank for the two-step partial and part correlations; ##, the cut-off similarity score; $$, the weight factor of the mixture similarity score; The numbers in bold and italic indicate the maximum for each of the peak alignment methods.**

Table S6. F1 score and AUC of each peak alignment method using Maximum distance for Data II.

		Cosine	Pearson's	Spearman's	Partial	Part
F1 (%)	PAS	61.09* (0.31)#	61.17 (0.30)	49.31 (0.25)	***61.58 (0.87)***	58.52 (0.85)
	Rank				***30***	5
	DW-PAS	***59.24 (0.36)***	59.16 (0.36)	56.71 (0.31)	59.23 (1.01)	57.59 (1.12)
	$K^{\$}$	***20***	20	10	20	20
	q**				50	3
	SW-PAD	59.26 (0.44)	59.10 (0.41)	55.62 (0.40)	***66.47 (0.84)***	57.86 (0.80)
	$\rho^{\#\#}$	0.93	0.93	0.3	***0.7***	0.1
	q				***50***	100
	PAM	61.48 (0.31)	61.51 (0.33)	59.29 (0.30)	***61.78 (0.91)***	60.19 (1.00)
	$w^{\$\$}$	0.05	0.05	0.1	***0.1***	0.5
	q				***30***	3
AUC (%)	PAS	84.53	84.55	72.55	***85.07***	83.16
	DW-PAS	***81.14***	81.14	79.64	81.00	80.23
	SW-PAD	77.42	77.81	77.18	***82.51***	75.31
	PAM	76.89	76.89	76.77	***77.45***	77.14

***, Mean (%); #, Standard error (%); $, the cut-off rank; **, the rank for the two-step partial and part correlations; ##, the cut-off similarity score; $$, the weight factor of the mixture similarity score; The numbers in bold and italic indicate the maximum for each of the peak alignment methods.**

Table S7. F1 score and AUC of each peak alignment method using Manhattan distance for Data II.

		Cosine	Pearson's	Spearman's	Partial	Part
**F1 (%)**	**PAS**	61.09* (0.31)#	61.17 (0.30)	49.31 (0.25)	***61.58 (0.87)***	58.52 (0.85)
	**Rank**				***30***	5
	**DW-PAS**	59.15 (0.34)	59.08 (0.35)	56.68 (0.33)	***59.15 (0.96)***	57.44 (1.03)
	k$	20	20	10	***20***	15
	**q****				***70***	3
	**SW-PAD**	59.54 (0.41)	59.38 (0.39)	56.32 (0.37)	***66.65 (0.77)***	57.96 (0.75)
	ρ##	0.93	0.93	0.3	***0.7***	0.1
	**q**				***50***	100
	**PAM**	61.48 (0.31)	61.49 (0.33)	59.27 (0.30)	***61.78 (0.91)***	60.19 (1.00)
	w$$	0.05	0.05	0.1	***0.1***	0.5
	**q**				***30***	3
**AUC (%)**	**PAS**	84.53	84.55	72.55	***85.07***	83.16
	**DW-PAS**	***81.66***	81.66	80.57	81.46	80.84
	**SW-PAD**	78.06	78.34	77.77	***82.90***	75.35
	**PAM**	76.87	76.87	76.79	***77.45***	77.18

***, Mean (%); #, Standard error (%); $, the cut-off rank; **, the rank for the two-step partial and part correlations; ##, the cut-off similarity score; $$, the weight factor of the mixture similarity score; The numbers in bold and italic indicate the maximum for each of the peak alignment methods.**

Table S8. F1 score and AUC of each peak alignment method using Canberra distance for Data II.

		Cosine	Pearson's	Spearman's	Partial	Part
**F1 (%)**	**PAS**	61.09* (0.31)#	61.17 (0.30)	49.31 (0.25)	***61.58 (0.87)***	58.52 (0.85)
	**Rank**				***30***	5
	**DW-PAS**	59.32 (0.33)	59.23 (0.34)	56.74 (0.32)	***59.52 (0.95)***	58.23 (1.02)
	K$	15	15	5	***20***	15
	**q****				***50***	15
	**SW-PAD**	59.09 (0.38)	58.86 (0.37)	54.83 (0.50)	***66.28 (0.63)***	57.65 (0.67)
	ρ##	0.93	0.93	0.3	***0.7***	0.1
	**q**				***50***	100
	**PAM**	61.21 (0.32)	61.21 (0.32)	59.42 (0.36)	***61.73 (0.87)***	59.72 (0.91)
	w$$	0.01	0.01	0.7	***0.1***	0.7
	**q**				***30***	3
**AUC (%)**	**PAS**	84.53	84.55	72.55	***85.07***	83.16
	**DW-PAS**	***83.45***	83.43	81.50	83.30	82.71
	**SW-PAD**	77.52	77.60	77.76	***82.74***	75.21
	**PAM**	80.78	80.80	79.22	***83.42***	82.76

***, Mean (%); #, Standard error (%); $, the cut-off rank; **, the rank for the two-step partial and part correlations; ##, the cut-off similarity score; $$, the weight factor of the mixture similarity score; The numbers in bold and italic indicate the maximum for each of the peak alignment methods.**
